# Photo-Induction and Automated Quantification of Reversible Mitochondrial Permeability Transition Pore Opening in Primary Mouse Myotubes

**DOI:** 10.1371/journal.pone.0114090

**Published:** 2014-11-25

**Authors:** Lionel Blanchet, Sander Grefte, Jan A. M. Smeitink, Peter H. G. M. Willems, Werner J. H. Koopman

**Affiliations:** 1 Department of Biochemistry, Radboud Institute for Molecular Life Sciences, Radboud University Medical Center, Nijmegen, The Netherlands; 2 Institute for Molecules and Materials, Analytical Chemistry/Chemometrics, Radboud University Nijmegen, Nijmegen, The Netherlands; 3 Centre for Systems Biology and Bioenergetics, Radboud University Medical Center, Nijmegen, The Netherlands; 4 Department of Paediatrics, Nijmegen Centre for Mitochondrial disorders, Radboud University Medical Center, Nijmegen, The Netherlands; University of Birmingham, United Kingdom

## Abstract

Opening of the mitochondrial permeability transition pore (mPTP) is involved in various cellular processes including apoptosis induction. Two distinct states of mPTP opening have been identified allowing the transfer of molecules with a molecular weight <1500 Da or <300 Da. The latter state is considered to be reversible and suggested to play a role in normal cell physiology. Here we present a strategy combining live-cell imaging and computer-assisted image processing allowing spatial visualization and quantitative analysis of reversible mPTP openings (“ΔΨ flickering”) in primary mouse myotubes. The latter were stained with the photosensitive cation TMRM, which partitions between the cytosol and mitochondrial matrix as a function of mitochondrial membrane potential (ΔΨ). Controlled illumination of TMRM-stained primary mouse myotubes induced ΔΨ flickering in particular parts of the cell (“flickering domains”). A novel quantitative automated analysis was developed and validated to detect and quantify the frequency, size, and location of individual ΔΨ flickering events in myotubes.

## Introduction

The mitochondrial permeability transition pore (mPTP) is a non-selective channel located in the mitochondrial inner membrane (MIM) and its opening (“permeability transition”) was first described 40 years ago [1]–[4]. In the open state, the mPTP allows ions and solutes up to a size of 1500 Da to passively diffuse over the MIM, leading to a rapid collapse of the highly inside-negative electrical potential (ΔΨ) across this membrane. The probability of mPTP opening is increased by elevated calcium concentrations in the mitochondrial matrix ([Ca^2+^]_m_), but other factors such as reactive oxygen species (ROS), pH, and ΔΨ also regulate this [5]. Permeability transition is involved in apoptotic and necrotic cell death, for instance during ischemia-reperfusion and muscular dystrophies due to collagen VI or laminin-2 deficiencies [6]–[8].

Currently, the molecular identity of the mPTP remains obscure. Although the adenine nucleotide translocase (ANT) and voltage-dependent anion channel (VDAC) were suggested as potential mPTP structural proteins, genetic studies disproved such a function for VDAC and revealed that ANT acts as a regulator of permeability transition [9]–[11]. Similarly, the mitochondrial phosphate carrier (PiC) was proposed as a mPTP structural protein although decreasing PiC expression up to 80% by RNA interference strategies did not affect mPTP opening [12]. Recent studies suggest that the mitochondrial F_o_F_1_-ATP synthase constitutes a structural component of the mPTP [13],[14]. A well-characterized mPTP modulator is the mitochondrial matrix protein Cyclophilin D (CypD), which increases the probability of Ca^2+^-dependent mPTP opening. The immunosuppressant cyclosporin A (CsA) is known to inhibit mPTP opening via CypD and thereby desensitizing the mPTP to Ca^2+^-stimulated opening [15]. This property makes CsA a widely used experimental tool to demonstrate involvement of mPTP opening in mitochondria-associated cellular phenomena.

Interestingly, during opening the mPTP can assume either a low- or a high-conductance state. In the low-conductance state the mPTP has a MW cut-off below 300 Da and thus only allows passage of small ions including H^+^ and Ca^2+^. Additionally, when in low-conductance mode, the mPTP opens transiently (“flickering”) and mitochondrial swelling is absent [16],[17]. In the high-conductance state the mPTP displays a much higher cut-off (below 1500 Da) and opening is permanent, resulting in sustained ΔΨ depolarization, mitochondrial swelling/rupture and cell death [16],[17].

Various methods have been described to study mPTP opening. For instance, permeability transition can be monitored in isolated mitochondria by quantifying the extent of mitochondrial swelling (measuring absorbance), mitochondrial Ca^2+^ retention capacity (CRC) or ΔΨ depolarization [15],[18],[19]. Although mitochondria are highly accessible by the above strategies, a major limitation of these techniques is the lack of a cellular context. This means that cytosolic factors that potentially regulate mPTP opening are absent. Moreover, isolation of mitochondria from tissues and cells significantly alters their structure, electrical connectivity and function [20]. In intact cells, permeability transition can be monitored using cationic fluorescent probes such as methyl (TMRM) or ethyl (TMRE) esters of tetramethylrhodamine, which accumulate in the mitochondrial matrix in a ΔΨ-dependent manner [21]–[24].

Although mPTP-dependent flickering of ΔΨ has been observed in studies of isolated mitochondria and intact cells [25]–[28], to the best of our knowledge no automated quantitative method for their combined spatial and temporal analysis exists. This precludes unbiased statistical comparison of reversible mPTP opening with respect to their spatiotemporal properties under varying experimental conditions. Here we present an integrated experimental and computational approach for automatic detection, spatial visualization and frequency analysis of ΔΨ depolarization and repolarization in primary mouse myotubes. Both the number of ΔΨ depolarizations/repolarizations were dramatically reduced by the mPTP inhibitor CsA, strongly suggesting that they reflect reversible mPTP opening. In contrast, inhibition of the F_o_F_1_-ATP synthase by oligomycin A increased the number of ΔΨ depolarizations/repolarizations. Both inhibitors reduced the spatial dimensions of the ΔΨ depolarization/repolarization region, suggesting that they affect the electrical connectivity of (the) mitochondrial (sub)network(s).

## Materials and Methods

### Animals and housing conditions

Mice were bred with a mixed 129/sv *x* C57BL/6 background. All animals received a standard rodent diet *ad libitum* and were maintained at 21.0°C and 60% humidity, and with a light/dark (12 h/12 h) cycle. All breeding and experiments were approved by the Animal Experimentation Committee at the Radboud University Medical Center, in accordance with Dutch laws and regulations regarding animal experimentation.

### Myofiber and primary myoblast isolation

Female mice (8–12 weeks) were sacrificed by decapitation and *extensor digitorum longus* (EDL) muscles were dissected. Individual myofibers were isolated as described in detail elsewhere [29]. EDL muscles were digested in 0.2% (w/v) Collagenase type I (Sigma Chemical CO, St Louis, MO, USA) in high-glucose (25 mM) Dulbecco's Modified Eagle's medium (DMEM-HG; Invitrogen HQ, San Diego, CA, USA)+1% penicillin/streptomycin (p/s, PAA Laboratories, Cölbe, Germany) for 1.5 hours. Using a heat-polished small-mouthed glass pipette, one-hundred myofibers were transferred to a new petri dish containing DMEM-HG+1% p/s. Then myofibers were washed and plated onto 6 wells-plates coated with 10× diluted Matrigel (Matrigel Basement Membrane Matrix, BD Bioscience, Bedford, MA, USA) and cultured with 6 ml DMEM-HG culture medium supplemented with 30% (v/v) fetal bovine serum (FBS, PAA Laboratories), 10% (v/v) horse serum (HS, PAA Laboratories), 1% (v/v) chick embryo extract (CEE, MP Biomedicals Europe, Illkirch, France), 10 ng/ml basic fibroblast growth factor (bFGF; Invitrogen), and 1% (v/v) p/s (PAA Laboratories). After three days, satellite cells grew out the myofibers, which were removed. Finally the satellite cells were trypsinized and pre-plated for 10 min in uncoated 6 wells-plate. Non-adherent satellite cells were transferred to a fresh Matrigel-coated 6 wells-plate, cultured for one week, and then used for the experiments.

### Primary myoblast cell culture

Coverslips (∅24 mm) were first coated with 20 µg/ml fibronectin (Roche Diagnostics GmbH, Mannheim, Germany) for one hour and subsequently with 10 µl Matrigel to create a “Matrigel-spot” for the cells. To induce fusion, 5,000 primary mouse myoblasts were seeded onto the Matrigel-spot in 10 µl DMEM-HG culture medium and after attachment (10 min) cultured with 3 ml DMEM-HG differentiation medium supplemented with 2% HS and 1% p/s (both PAA Laboratories) for 3 days at 37°C (95% air, 5% CO_2_).

### Microscopy imaging of TMRM-stained mouse myotubes

To visualize mitochondria and detect ΔΨ changes, myotubes were loaded with 30 nM tetramethylrhodamine methyl ester (TMRM, Invitrogen) in DMEM-HG differentiation medium for exactly 25 minutes. Under these conditions TMRM operated in non-quenching mode. Then cells were washed and transferred to a HEPES-Tris (HT) medium (132 mM NaCl, 4.2 mM KCl, 1 mM CaCl_2_, 1 mM MgCl_2_, 5.5 mM D-glucose and 10 mM HEPES, pH 7.4). The coverslips were mounted in an incubation chamber and placed on the stage of an inverted microscope (Axiovert 200 M, Carl Zeiss, Germany). For noise analysis, empty coverslips were mounted on the microscope and HT-buffer containing different concentrations of TMRM (0–80 nM) was used. TMRM was excited at 540 nm using a monochromator (Polychrome IV, TILL Photonics, Gräfelfing, Germany) and a Zeiss 40×/1.3 NA Plan NeoFluar objective. Fluorescence light was directed using a 560DRLP dichroic mirror (Omega Optical Inc, Brattleboro, VT, USA) and a 565ALP emission filter (Omega Optical Inc.) onto a CoolSNAP HQ monochrome CCD-camera (Roper Scientific, Vianen, The Netherlands). The microscopy hardware was controlled using Metafluor 6.0 software (Universal Imaging Corporation, Downingtown, PA, USA) and images were recorded every 2 seconds for a period of 20 minutes using a 300 ms image acquisition time.

### Electron microscopy

5,000 mouse primary myoblasts were seeded onto the Matrigel-spot in 10 µl DMEM-HG culture medium and after attachment (10 min) cultured with 3 ml DMEM-HG differentiation medium for 3 days at 37°C (95% air, 5% CO_2_). Then myotubes were washed with PBS and fixed in 2% glutaraldehyde buffered with 0.1 M sodium cacodylate pH 7.4. After 1 hour of fixation the myotubes were post-fixed in 1% osmium tetroxide in Palade's buffer pH 7.4 with 1% potassium hexacyanoferrate (III)-trihydrate. After dehydration in ethanol and propylene oxide, tubes were embedded in Epon. Semi-thin, 1 µm thick transverse sections were stained with 1% toluidine blue. Ultrathin sections were stained with uranyl acetate and lead citrate and examined in a JEOL TEM1010 transmission electron microscope.

### Data analysis

Automated image processing and analysis were carried out using Image Pro Plus 6.3 software (Media Cybernetics, Silver Spring, MD, USA). Additional data processing, visualization and statistical analysis were performed using custom scripts written in Matlab 6.1 (Mathworks, Natick, Massachusetts, U.S.A.). Unless stated otherwise, data is presented as mean ± SD (standard deviation). The p-values reported in the results section correspond either to a two-way ANOVA (with two factors: manual vs. automated analysis and CsA treated vs. untreated) or one way ANOVA (1 factor with three levels: untreated, CsA or OLI treated cells). In the latter case, the ANOVA was followed by a Tukey *post-hoc* procedure.

## Results

### Photo-induced ΔΨ flickering in primary mouse myotubes

**Figure 1:**
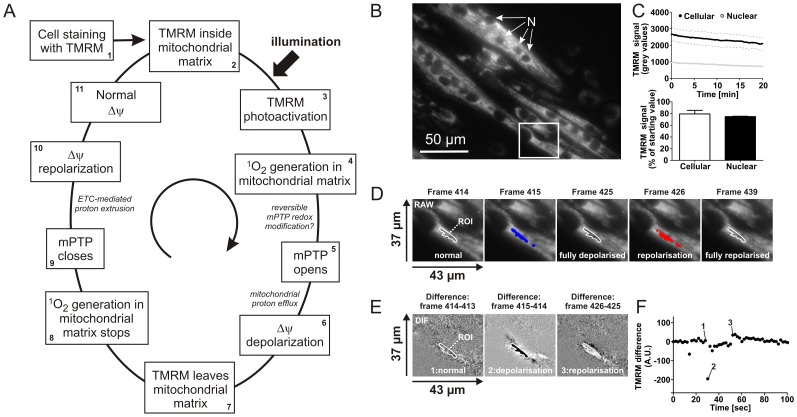
Photo-induction of ΔΨ flickering in TMRM-stained primary mouse myotubes. (**A**) Proposed mechanism underlying ΔΨ flickering in TMRM-stained cells [28]. Following TMRM staining (#1) and its accumulation in the mitochondrial matrix (#2) cells are illuminated leading to photo-induced generation of singlet oxygen (^1^O_2_) within the mitochondrial matrix (#4). As a consequence opening of the mPTP is stimulated (#5) leading to ΔΨ depolarization (#6) and TMRM efflux from the mitochondrial matrix (#7). The latter prevents photo-induced ^1^O_2_ generation in the matrix (#8), resulting in mPTP closure (#9), ΔΨ repolarization mediated by action of the mitochondrial electron transport chain (ETC; #10) and subsequent ΔΨ normalization (#11) after which the next depolarization/repolarization cycle can start. (**B**) Typical example of TMRM-stained myotubes. Nuclei are marked by ‘N’. The region of interest (ROI) depicted in panel D is indicated by a rectangle. (**C**) Upper panel: Time dependence of the average cellular and nuclear TMRM fluorescence intensity (N = 5 myotubes) during 20 min of recording. Lower panel: average cellular and nuclear TMRM fluorescence intensity after 20 min of recording (N = 5 myotubes). (**D**) Typical time-frames illustrating the localized disappearance (pixels colored blue by thresholding) and reappearance (pixels colored red by thresholding) of TMRM fluorescence intensity. (**E**) Difference (DIF) images calculated from the recorded images (see results for details) depicting cellular regions displaying ΔΨ depolarization (black pixels) and ΔΨ repolarization (white pixels). (**F**) Time dependent signal changes in the DIF images for the ROI marked in the first image. Numerals correspond to the images in panel E.

Current evidence supports a mechanism in which TMRM illumination stimulates the cyclic photo-generation of mitochondrial singlet oxygen molecules (^1^O_2_), which increase the probability of mPTP opening [28]. As a consequence, a cycle of ΔΨ depolarization and repolarization is induced leading to cyclic mitochondrial TMRM efflux and re-uptake (Fig. 1A). To monitor this cycle, primary myotubes were stained with the fluorescent cation TMRM and visualized using an epifluorescence video microscope (Fig. 1B). TMRM partitioning between the cytosol and mitochondrial matrix primarily depends on the magnitude of the electrical membrane potential (ΔΨ) across the mitochondrial inner membrane (MIM). Fields of views (FOVs) were chosen in such a way that they mainly contained myotubes to prevent interference from myoblasts. Myotubes are easily identified because they contain multiple nuclei (Fig. 1B; indicated by “N”), whereas myoblasts do not. For each time lapse recording (“image stack”) 600 images were routinely acquired using an interval of 2 s and an illumination time of 300 ms. The recorded images had a bit-depth of 12 and were converted to TIFF format for subsequent visualization and processing. Visual inspection highlighted the presence of localized TMRM intensity fluctuations (“flickering”; Movie S1), which typically were initiated within 20 seconds after the start of recording (*i.e.* after 10 images were acquired). The total integrated cellular and nuclear TMRM signal both slowly decreased (Fig. 1C) to 75–80% with respect to their initial TMRM signal. This suggests that part of the TMRM intensity is lost due to photo bleaching. The fact that both the mitochondrial and nuclear TMRM intensity decreased at a similar rate indicates that the TMRM “flickering” is fully reversible and not associated with permanent TMRM repartitioning (*i.e.* sustained ΔΨ depolarization).

### Visualization of ΔΨ flickering and its association with mPTP opening

**Figure 2:**
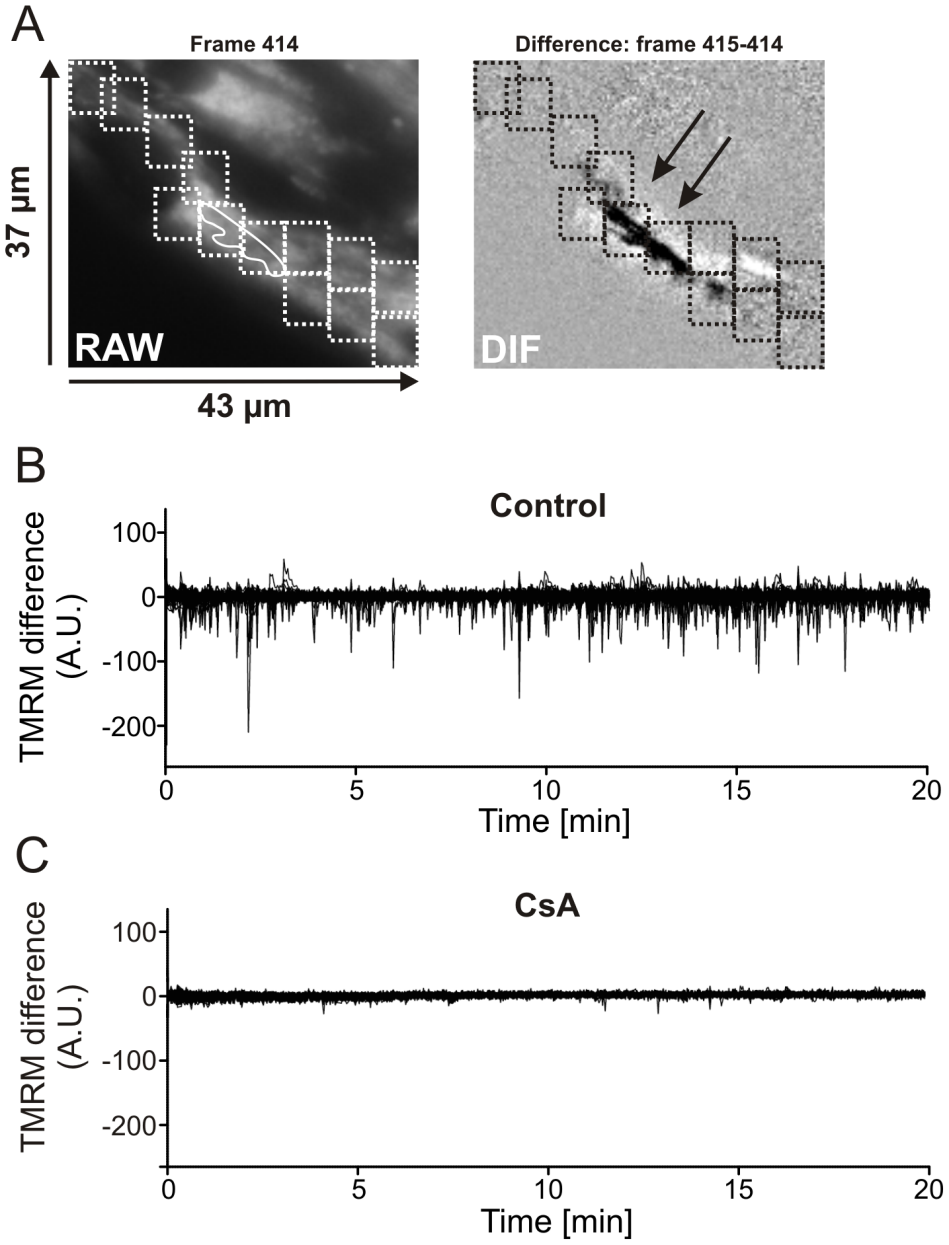
Manual analysis of ΔΨ flickering temporal properties. (**A**) A typical example of the position of identical rectangular regions of interest (ROIs; dotted rectangles) along the myotube in the raw frame (left panel) and DIF frame (right panel). The two black arrows indicate two ROIs detecting the same depolarization. (**B**) Variation of signal intensity in the DIF frames of ∼110 ROIs in one cell during 20 minutes. (**C**) Similar to panel B but now for ∼100 ROIs in 2 myotubes treated with the mitochondrial permeability transition pore inhibitor cyclosporine A (CsA; 5 µM, 2 h).

A typical depolarization (blue) and repolarization event (red) for a specific region of interest (ROI) are shown in figure 1D. To better visualize the TMRM intensity changes and minimize interference by TMRM photo bleaching, difference images were calculated between each image (N) and its preceding image (N-1) in the image stack. This yields a new image stack (“DIF”) in which ΔΨ depolarizations and repolarizations appear as regions of dark and light pixels, respectively (Fig. 1E). This allows graphical visualization of the average signal changes within a ROI as a function of time (*e.g.* Fig. 1F). To demonstrate that these depolarization and repolarization events arise from mPTP opening, a manual analysis was performed on DIF stacks recorded from control myotubes and myotubes treated with the mPTP-inhibitor Cyclosporin A (CsA; 5 µM, 2 h) (Fig. 2). To allow faithful event detection, an ROI of identical size was used and placed along the whole myotube using the raw frames (see Fig. 2A left panel for a part of a typical myotube). ROI size was empirically chosen in such a way that it was large enough to minimize the number of ROIs required, but small enough to still pick up low magnitude intensity changes. Then these ROIs were used in the DIF stack (Fig. 2A; right panel) for temporal intensity analysis of the complete myotube. In figure 2B, traces of ∼110 ROIs of one control myotube are shown. Clearly, many sharp downward peaks (corresponding to ΔΨ depolarization) were observed in control myotubes. Additionally, lower amplitude upward peaks (corresponding to ΔΨ repolarization within the same ROI) were also observed. Visual inspection of the DIF stack confirmed the ROI-dependent presence of ΔΨ depolarization (dark pixels) and ΔΨ repolarization (light pixels). The same strategy with equal ROIs was used on myotubes pre-treated with CsA. CsA pre-treatment greatly reduced the occurrence of depolarization and repolarization events (Fig. 2C; ∼100 ROIs of two myotubes). This strongly suggests that the observed ΔΨ flickering arises from reversible mPTP openings. Although quantitative, a major drawback of manual ROI analysis is its time-consuming nature. Moreover, due to the size of the ROIs and their (sometimes overlapping) positioning to cover the entire cell, some events can be simultaneously detected within multiple ROIs (indicated by the black arrows in Fig. 2A right panel), thereby over-estimating their number. To address these issues we developed an automated analysis strategy.

### Evaluation of the shot noise influence

**Figure 3:**
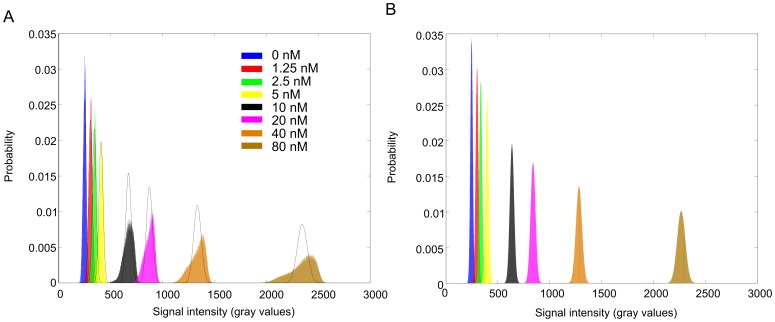
Histogram of fluorescence intensities of TMRM at different concentrations. (**A**) Fluorescence intensity histograms for TMRM solutions, T = 25°C. Bars – experimental data, solid lines – theoretical Poisson distributions for the experimental mean. Probability is the number of pixels with a given fluorescence intensity divided by the total number of pixels. (**B**) Difference intensity histograms for TMRM solutions. Note that the distributions are displayed at the same position as the original signal for clarity reasons, as the difference signals are normally centered on zero.

To demonstrate that the changes in TMRM fluorescence intensity is a result of a biological process (i.e. ΔΨ flickering) and not due to instrumental background noise, the influence of shot noise was estimated using a protocol similar proposed by *Blinova et al.* [30]. The fluorescence signal of TMRM solutions at different concentrations (0 to 80 nM) was recorded using identical microscopic settings as for the cells. The theoretical Poisson distribution and experimental distribution were compared, as shown in Figure 3. At high TMRM concentration, the observed noise deviated from the theoretical distribution, likely due to uneven illumination across the FOV and temperatures effects. However, when using the difference signal as calculated in our protocol for ΔΨ analysis (see above) the noise distribution was normally distributed and displayed a limited spread across the range of TMRM concentrations used. This indicates that the changes in TMRM fluorescence intensity are a consequence of a biological process in the myotubes and not a consequence of instrumental background noise.

### An automated protocol for quantification of ΔΨ flickering frequency and domain size

**Figure 4:**
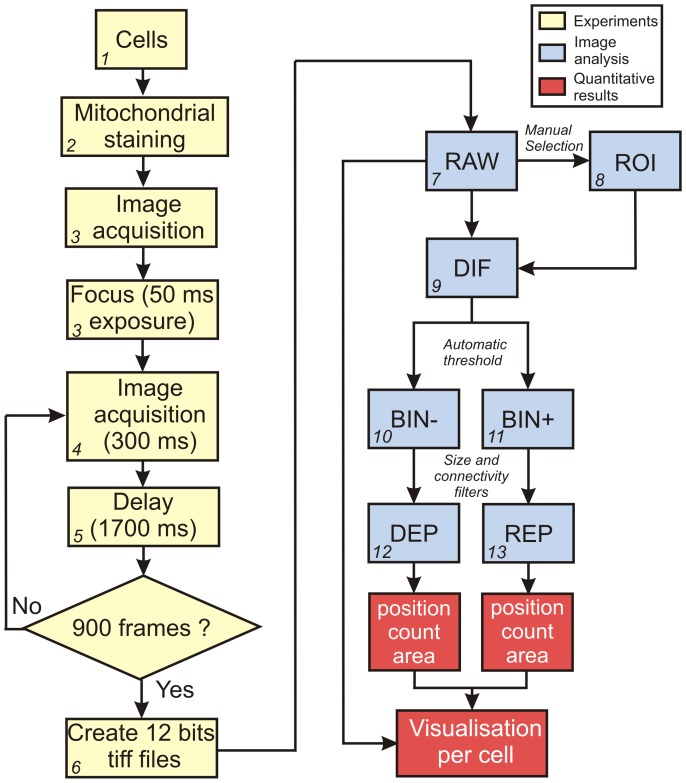
Combined experimental and computational strategy for quantitative spatiotemporal analysis of ΔΨ flickering. Flow scheme visualizing the sequential experimental strategy (yellow), image processing and quantification (blue) and the quantitation and visualization of numerical results (red). Additional details regarding this strategy are provided in the results section.

**Figure 5:**
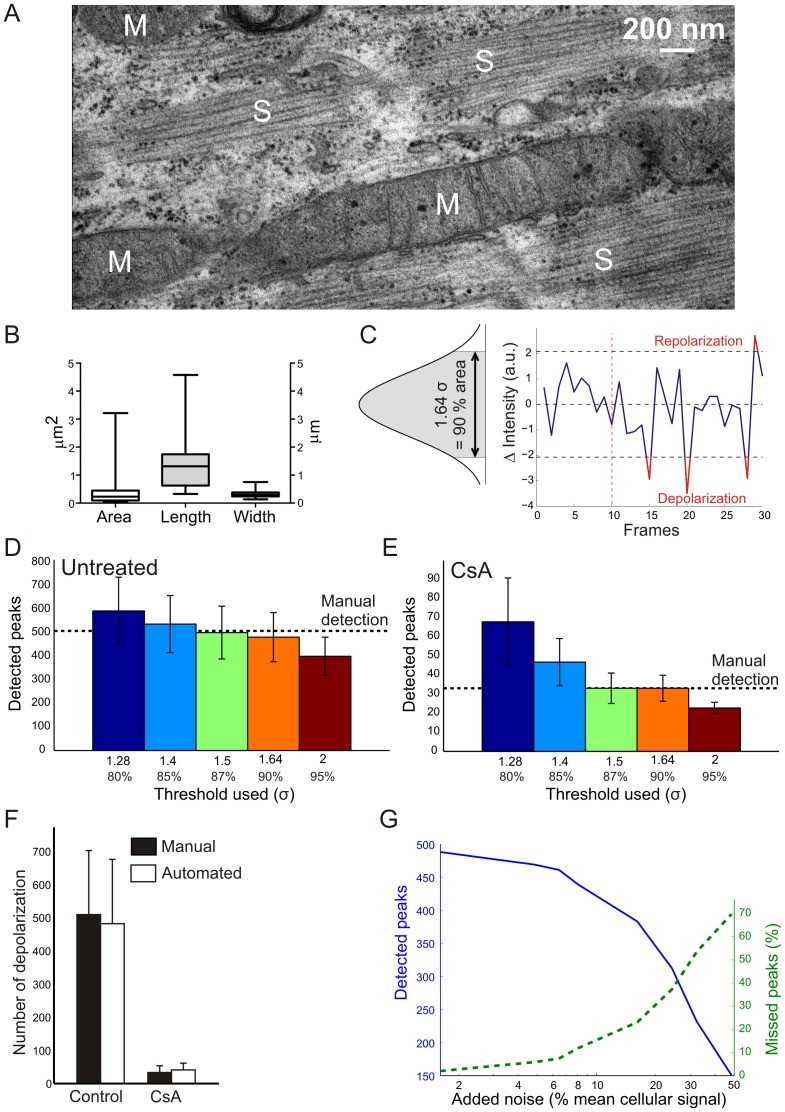
Optimization and validation of the automated algorithm for spatiotemporal analysis of ΔΨ flickering. (**A**) Transmission electron microscopy (TEM) picture of mitochondria in primary mouse myotubes. M indicates mitochondria and S indicates sarcomeres. (**B**) Box and whisker plot showing the median, minimum and maximum value, and the 1^st^ and 3^rd^ quartile of the mitochondrial (N = 31) area, length and width in myotubes based on TEM. (**C**) Process to define the threshold used to detect mitochondrial depolarizations and repolarizations. Different thresholds, 1.28 (80% certainty), 1.4 (85% certainty), 1.5 (87% certainty), 1.64 (90% certainty), and 2 (95.5% certainty) times σ, were used and their corresponding numbers of depolarizations were compared to manual analysis of untreated (**D**) and CsA-treated (**E**) myotubes. (**F**) The number of mitochondrial depolarizations detected by the optimized computer-assisted analysis are comparable to the manual analysis in control (N = 8 in 3 experimental replicates) and CsA-treated (N = 7 in 3 experimental replicates) myotubes. The CsA treatment led to a significant difference (p≤0.001) while manual and automated analysis cannot be distinguished statistically (p = 0.454) (**G**) Addition of noise to the movie to define the robustness of the computer-assisted analysis.

A flow scheme of the automated strategy is presented in Figure 4. The experimental part (yellow) and image analysis part (blue) including the creation of the DIF stack were already described above. For automated analysis we manually defined a ROI that encompassed a single myotube. We aimed to design an algorithm compatible with the spatial resolution (*i.e.* considering the smallest observable mitochondrial size) and quality (*i.e.* signal-to-noise ratio) of the microscopy images. Figure 5A depicts a typical electron microscopy (EM) picture of a mitochondrion in a mouse myotube. Quantification of mitochondrial dimensions in multiple EM images yielded average values for mitochondrial area (0.460 µm^2^), length (1.6 µm) and diameter (0.333 µm) (Fig. 5B, N = 31). A reticular was used to calibrate the microscopy images and showed that these dimensions are within the same range as the lateral (XY) dimensions (*i.e.* 0.3 µm). This means that, in theory, a single pixel could correspond to a small mitochondrial object. However, a single pixel intensity change could also be reflecting a noise-related artifact. Considering mitochondrial length we assumed that mitochondrial objects are longer than 3 pixels (*i.e.* 0.9 µm). It was further assumed that individual mitochondria can be randomly oriented so a 3×3 square region with an area of 0.81 µm^2^ was used. Although this area exceeds that of an average mitochondrion (Fig. 5B), average mitochondrial length is larger than 3 pixels. Therefore, we defined a mitochondrial object as a region with a size larger than 3×3 pixels displaying TMRM flickering.

The next challenge is to carry out a threshold operation on the DIF stack that faithfully separates “real” signal changes due to ΔΨ flickering and signal changes due to random noise. To automatically determine the optimal threshold value, a decision algorithm was applied based on the assumption that ΔΨ flickering is absent during the first 10 frames of the stack. This assumption was proven valid by visual inspection of the acquired DIF stacks. Next, the fluctuations in the DIF stack signal during the first 10 frames were analyzed to calculate the standard deviation (SD) value (σ), which was used to define various threshold values. For example, a value of 1.64 times σ corresponded to 90% of the area of a Gaussian distribution (Fig. 5C), meaning that signals in the DIF stack exceeding this value can be considered to represent ΔΨ flickering events with 90% certainty. Using this threshold, three depolarization events and one repolarization event were highlighted in the partial recording depicted in Figure 5C. Obviously, the number of detected ΔΨ flickering events decreases with increasing threshold values. To find the optimal threshold value we determined the number of ΔΨ depolarization peaks as a function of σ in untreated (Fig. 5D) and CsA-treated myotubes (Fig. 5E). Comparing this number with that obtained by manual analysis (dotted horizontal lines), the value 1.64 (90% confidence) and not 1.5 (87% confidence) times σ was chosen as the most optimal threshold, since the former value gives a higher confidence. By applying this optimal threshold value, two new image stacks were generated from each DIF stack (Fig. 4). One with below-threshold signal changes (BIN−; reflecting ΔΨ depolarizations) and another with above-threshold signal changes (BIN+; reflecting ΔΨ repolarizations).

In addition to the temporal properties of the ΔΨ flickering events, we also wanted to quantify their spatial properties in the BIN− and BIN+ images. Our results (*e.g.* Fig. 1 and 2) demonstrate that ΔΨ depolarization is relatively fast and associated with simultaneous intensity changes in regions with a size of multiple pixels (“flickering domains”). As explained above we first discarded flickering domains that were smaller than 9 contiguous pixels (forming a 3×3 square region) from our analysis. Moreover, when closely juxtaposed ROIs displayed synchronized ΔΨ flickering events we assumed that they constituted part of the same flickering domain. The latter was included in our algorithm in that a 15 pixels wide radius (0.45 µm), around each flickering domain was scanned for the presence of another domain with synchronous flickering. If so, this domain was considered part of the same flickering domain. Application of the threshold and size criteria on the BIN− and BIN+ stacks yields two new binary stacks (Fig. 4) displaying the position of ΔΨ depolarizations (DEP) and repolarizations (REP). The result of this automated strategy was validated by manual analysis. For this purpose multiple DIF stacks of control myotubes (N = 8 in 3 experimental replicates) and CsA-treated (N = 7 in 3 experimental replicates) myotubes were visually inspected frame-by-frame; each ΔΨ flickering and its position was manually scored. The localization (data not shown) and number (Fig. 5F) of ΔΨ depolarizations obtained by manual and automated analysis was identical and the latter was also significantly reduced by CsA treatment (p≤0.001) (Fig. 5F). Finally, we evaluated the noise-sensitivity of our algorithm by artificially adding different amounts of (white) Gaussian noise to each image in a typical stack. Subsequent application of the analysis algorithm (Fig. 4) revealed that noise addition increased the percentage of missed peaks (Fig. 5G). This result demonstrates that our strategy requires image stacks with a sufficiently high signal-to-noise ratio.

### Spatiotemporal properties of ΔΨ flickering in control myotubes

**Figure 6:**
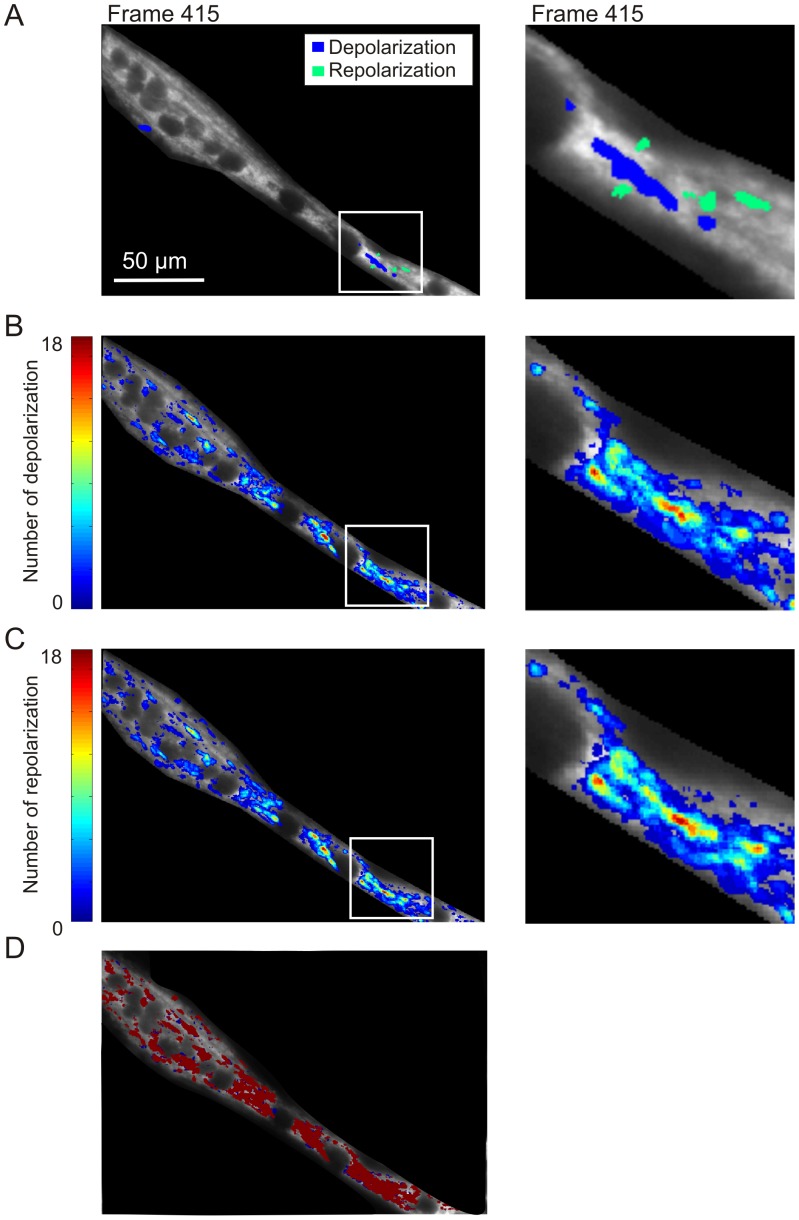
Spatiotemporal properties of ΔΨ flickering in primary mouse myotubes. (**A**) Typical image of a TMRM-stained myotube (left panel). (**B**) Image obtained by summing all images within the DEP stack, reflecting the total number and position of the ΔΨ depolarizations (left panel). The image was color coded based upon the number of depolarizations. (**C**) Similar to B but now using the REP stack to reflect the total number and position of the ΔΨ repolarizations. The rectangle in A, B, and C indicates the region of interest (ROI) magnified in the corresponding panels to the right. **D**) Co-localization of the ΔΨ depolarizations and repolarizations observed in panel B and C. See results for details.

Applying our analysis protocol (Movie S2) revealed that ΔΨ depolarizations occur in specific regions (Fig. 6A; blue). Depolarizations were typically paralleled by simultaneous ΔΨ repolarizations (or even hyperpolarizations) within adjacent ΔΨ flickering domains (Fig. 6A; green). This suggests that TMRM released from a depolarizing domain (blue) can be taken up by mitochondria in a nearby hyperpolarizing domain (green). By summing all images within the DEP or REP stack for a given myotube, two images can be calculated to visualize the total number of depolarizations (Fig. 6B; calculated from DEP stack) and repolarizations (Fig. 6C; calculated from REP stack). These images also reveal the position and domain size of the flickering domains. Multiple events were observed within the same domains, suggesting that mitochondria within these domains are electrically coupled (Fig. 6B; red). Certain regions within the myotube displayed more flickering events than other, suggesting the existence of ΔΨ flickering ‘hotspots’. In contrast, ΔΨ flickering was not detected in other regions of the myotubes during the time course of the experiment (Fig. 6; noncolorized pixels). Comparing the data obtained for ΔΨ depolarizations (Fig. 6B) and repolarizations (Fig. 6C) revealed highly similar patterns. Furthermore, we estimated the co-localization of both types of events as the ratio between the number ΔΨ depolarizations and repolarizations per pixel, as shown in Fig. 6D. The average co-localization, as reflected by the Manders coefficient [31], equaled 0.93. This indicates that the large majority of the observed events reflect reversible ΔΨ flickering that represents mPTP opening.

### Spatiotemporal properties of ΔΨ flickering in myotubes treated with CsA and oligomycin A

**Figure 7:**
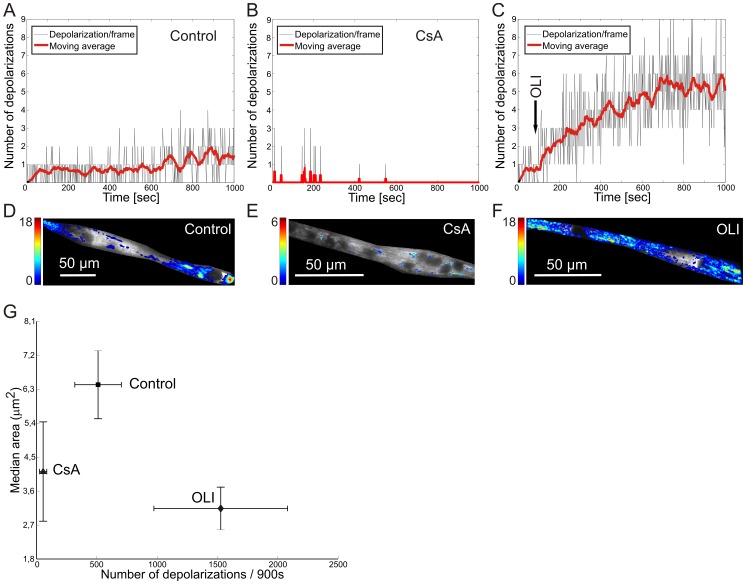
Inhibition of the F_o_F_1_-ATP synthase affects the spatiotemporal properties of ΔΨ flickering. (**A**) Time dependence of the number of depolarizations (black line) in a control myotube. A moving average of the signal was calculated at every time-point with a 2 second interval for 1000 seconds (red line) to facilitate visual inspection. (**B**) Similar to panel A but now with CsA treatment. (**C**) Similar to panel A but now with acute addition (arrow) of the F_o_F_1_-ATP synthase inhibitor oligomycin A (OLI; 10 µM). (**D**) Image obtained by summing all images within the DEP stack of the experiment depicted in panel A, reflecting the total number and position of the ΔΨ depolarizations. (**E**) Similar to panel D but now for the experiment depicted in panel B. (**F**) Similar to panel D but now for the experiment depicted in panel C. (**G**) Effect of CsA (N = 7) and OLI (N = 3) on the number of ΔΨ depolarizations (x-axis) and median area of the depolarizing region (y-axis). Both the median area and the frequency of OLI-treated myotubes are significantly different from controls and CsA-treated myotubes (in both cases p<0.01).

Recent evidence suggests that the mitochondrial F_o_F_1_-ATP synthase (complex V or CV) constitutes (part of) the mitochondrial permeability transition pore [13],[14]. It was further observed that CypD, which mediates CsA-induced mPTP inhibition, interacts with the OSCP (oligomycin-sensitivity conferring protein) subunit of CV. To demonstrate whether inhibition of CV activity affects photo-induced ΔΨ flickering we analyzed mouse myotubes in the absence and presence of the specific CV-inhibitor oligomycin A (OLI; 10 µM). Interestingly, relative to untreated myotubes, CsA treatment greatly diminished ΔΨ flickering frequency as expected. However, acute OLI treatment 3-fold increased ΔΨ flickering frequency within 15 min (Fig. 7A–C), suggesting that short-term CV inhibition acutely stimulates ΔΨ flickering. The increase of the ΔΨ flickering upon OLI treatment could not be reduced by CsA. During OLI treatment, the increase in flickering frequency was paralleled by complete disappearance of the ‘hotspots’ observed in untreated cells (Fig. 7D) and depolarizations occurred throughout the myotube (Fig. 7F). The fact that the median area of the detected flickering domains was ∼70 pixels, *i.e.* 6.3 µm^2^ in untreated myotubes (Fig. 7G), suggests that such a domain consists of multiple mitochondria displaying synchronized ΔΨ flickering or an electrically connected mitochondrial (sub)network. Inter-mitochondrial electrical coupling appears to be lost in OLI-treated cells. The size of the ΔΨ flickering domains was 2-fold reduced in OLI-treated cells (Fig. 7G). CsA-induced inhibition of ΔΨ flickering was also associated with a 2-fold reduction in size of the ΔΨ flickering domains.

## Discussion

This study presents an integrated experimental and computational approach allowing detection and spatiotemporal analysis of light-induced ΔΨ flickering events in TMRM-stained primary mouse myotubes. After validation, this strategy was applied to demonstrate that ΔΨ flickering is associated with mPTP opening.

### Photo-induction of ΔΨ flickering in TMRM-stained primary mouse myotubes

We previously discussed that TMRM is well suited for ΔΨ quantification given its low toxicity, fast equilibration kinetics across the MIM and low a-specific binding to mitochondrial membranes [32]. However, it is important to realize that mitochondrial accumulation of this dye above a certain concentration leads to autoquenching; making ΔΨ to appear depolarized [33]. However, acute application of the mitochondrial uncoupler FCCP (p-trifluoromethoxy carbonyl cyanide phenyl hydrazine) induced an immediate decrease in mitochondrial TMRM fluorescence (data not shown). Hence TMRM autoquenching is absent under our experimental conditions. Evidence was provided that illumination of TMRM and subsequent ^1^O_2_ formation drive mPTP opening [28],[34]. Compatible with this ^1^O_2_ formation we observe TMRM photobleaching in our experiments. However this photobleaching is not associated with changes in TMRM mitochondrial/nuclear distribution ratio, irreversible ΔΨ depolarization or aberrations in cell shape. This finding agrees with previous results demonstrating that TMRM illumination does not release toxic amounts of ^1^O_2_ within the mitochondrial matrix space [35].

In principle, a sudden decrease in TMRM intensity can also result from mitochondria moving out of focus instead of ΔΨ depolarization. To address this potential issue a ΔΨ-insensitive mitochondrial co-stain could be applied to normalize the number and size of ΔΨ depolarizations/repolarizations. In this study we refrained from using a second mitochondrial stain for several reasons: (i) it might affect mitochondrial physiology, possibly altering ΔΨ and intra-mitochondrial TMRM concentration, and (ii) it might act as a photosensitizer thereby likely altering experimental settings (*e.g.* illumination time or TMRM loading concentration). However, in our experiments the number of ΔΨ depolarizations and repolarizations are equal (Manders overlap coefficient of 0.93) making it unlikely that the observed ΔΨ flickering results from mitochondrial movement.

ΔΨ flickering activity is virtually abolished by CsA, supporting the conclusion that this flickering represents reversible mPTP opening events. With respect to mitochondrial CRC, a previous study suggests that this parameter increases with TMRM irradiation times up to 30 s (suggesting reduced mPTP opening) and subsequently decreases (suggesting increased mPTP opening) for a TMRM irradiation time >30s [34]. These results were obtained using isolated mouse liver mitochondria with a fully inhibited electron transport chain (ETC), which prevents ΔΨ repolarization. However, we also observed a slight increase in mPTP opening frequency at longer illumination times (*e.g.* Fig. 7A).

### A method for quantitative analysis of reversible ΔΨ flickering

Mitochondrial ΔΨ flickering was previously demonstrated in isolated mitochondria and intact cells [25]–[28]. However, quantitative analysis relied on manual analysis by placing small ROIs over (parts of) single mitochondria in different cell-types, which is time-consuming and might lead to biased quantitative analysis. More importantly, this method is susceptible to mitochondrial movement and focus drift. The novel method presented here addresses these points in myotubes and can also be more easily implemented in high-content (HCS) and/or high-throughput (HTS) microscopy screening strategies. In essence, our algorithm consists of calculating a stack of difference images (DIF) from an image stack (RAW) obtained by fluorescence microscopy. This approach has already been used before to visualize ΔΨ depolarization in complete astrocytes [26]. However, our method is able to automatically detect ΔΨ depolarizations and repolarizations in regions of a myotube and provide information about their spatial localization, area, and frequency. This requires a proper mitochondria-specific fluorescence signal to allow faithful discrimination between this organelle and the remainder of the cell. From the DIF stack two new image stacks (DEP and REP) are calculated by applying an automatically determined threshold operation. In this case, the optimum is to use 1.64 σ (equaling 90% of the area of a normal distribution) in the first 20 seconds of the experiments. Other options seem reasonable based on the results provided in figures 5D and 5E. In this sense, a value of 1.5 σ (reflecting 87% of the area of a normal distribution) yielded similar results. However, here we chose to use 1.64 σ since this value best matched the manual analysis while providing the highest (90%) confidence. The DEP and REP stacks are then used for numerical analysis of ΔΨ depolarization frequency and domain size (DEP stack) and ΔΨ repolarization frequency and domain size (REP stack). It was previously discussed that mPTP opening can induce full ΔΨ depolarization within 5 ms [28]. This time period is much smaller than our image illumination time (300 ms) and acquisition interval (2 s). However, following mPTP closure ΔΨ is restored by ETC action, as reflected by a slowly increasing mitochondrial TMRM fluorescence (data not shown) [28]. In this way, our algorithm is still able to reliably detect ΔΨ depolarizations. Quantitative comparison indicates that the numbers of detected ΔΨ depolarizations and ΔΨ repolarizations are identical, meaning that both types of events are reliably detected. In theory, the fact that our algorithm reported no ΔΨ flickering events in subcellular regions of control myotubes could be due to: (i) that these events are truly absent, (ii) that they originate from mitochondrial objects smaller than 3×3 pixels that are excluded from the analysis, or (iii) given the axial dimensions of the myotubes, mitochondrial objects might be out of focus. When treated with OLI, the algorithm reported an increased number of ΔΨ flickering events that originated from ΔΨ flickering domains with a substantially reduced size. This observation argues against mitochondria in control cells being so small that they evade detection. OLI-treated cells also displayed ΔΨ flickering domains throughout the complete myotube. This result argues against mitochondrial objects being out of focus. In the light of the above, we conclude that our algorithm reliably detects ΔΨ flickering events and that the latter are restricted to subcellular ΔΨ flickering domains in control myotubes.

### Cyclosporin A and oligomycin A affect the spatiotemporal properties of reversible mPTP openings associated with ΔΨ flickering

Our results demonstrate that ΔΨ flickering frequency is reduced by CsA, but increased by OLI. In contrast, both inhibitors reduced the size of ΔΨ flickering domains suggesting that they both reduce the electrical connectivity of (the) mitochondrial (sub)network(s). This means that a reduced mitochondrial electrical connectivity can be associated with either a decreased or increased mPTP opening frequency. Interestingly, a previous study demonstrated that mitochondrial matrix volume and CRC were reduced when isolated mitochondria were placed in a medium with low osmolarity (250 mOsM) whereas mitochondrial volume and CRC were increased in a medium with high osmolarity (400 mOsM) [36]. This suggests that a reduction in mitochondrial matrix volume inhibits Ca^2+^-induced mPTP opening. Therefore, the CsA-induced inhibition of mPTP opening observed in this study might be (partially) mediated by a reduction in mitochondrial size, compatible with smaller ΔΨ flickering domains.

Recent studies support the idea for a structural role of F_O_F_1_-ATP synthase in mPTP opening and in particular the c-subunit [13],[14],[37]. Oligomycin A, a potent inhibitor of the F_O_F_1_-ATP synthase, was shown to inhibit mPTP opening and TNF-α/ementine-induced cell death in HeLa cells [38]. Surprisingly, acute OLI treatment dramatically increased ΔΨ flickering frequency, reduced the size of ΔΨ flickering domains and triggered ΔΨ flickering events throughout the entire myotube. Although our aim and method is not to induce terminal depolarization and apoptosis, the observed phenomenon might lead eventually to apoptosis. However, OLI is able to increase the rate of apoptosis in eosinophils, suggesting that the effect of OLI could depend on the cell type used [39].

In conclusion, a novel quantitative automated analysis was developed to detect and quantify the frequency, size, and location of individual ΔΨ flickering events in myotubes as a consequence of mPTP opening.

## Supporting Information

Movie S1 **Typical movie of TMRM-stained myotubes.** In the myotubes (similar to figure 1B) several localized disappearances (ΔΨ depolarizations) and reappearances (ΔΨ repolarizations) of TMRM fluorescence intensity can be observed.
(WMV)

Movie S2 **Application of the analysis protocol on a TMRM-stained myotube.** The automated analysis protocol detects many ΔΨ depolarizations (pixels colored blue) and repolarizations (pixels colored green) in the myotube. The automated analysis protocol also calculates for each frame the number (# flashes) and size of the ΔΨ depolarizations (green line) and repolarizations (blue line), which are shown in two separate graphs.
(WMV)

## References

[1] Hunter DR, Haworth RA, Southard JH (1976) Relationship between configuration, function, and permeability in calcium-treated mitochondria. J Biol Chem 251:5069–5077. 134035

[2] Hunter DR, Haworth RA (1979) The Ca2+-induced membrane transition in mitochondria. I. The protective mechanisms. Arch Biochem Biophys 195:453–459. 383019 10.1016/0003-9861(79)90371-0

[3] Haworth RA, Hunter DR (1979) The Ca2+-induced membrane transition in mitochondria. II. Nature of the Ca2+ trigger site. Arch Biochem Biophys 195:460–467. 38751 10.1016/0003-9861(79)90372-2

[4] Hunter DR, Haworth RA (1979) The Ca2+-induced membrane transition in mitochondria. III. Transitional Ca2+ release. Arch Biochem Biophys 195:468–477. 112926 10.1016/0003-9861(79)90373-4

[5] Halestrap AP (2009) What is the mitochondrial permeability transition pore? J Mol Cell Cardiol 46:821–831. 19265700 10.1016/j.yjmcc.2009.02.021

[6] Halestrap AP, Pasdois P (2009) The role of the mitochondrial permeability transition pore in heart disease. Biochim Biophys Acta 1787:1402–1415. 19168026 10.1016/j.bbabio.2008.12.017

[7] Irwin WA, Bergamin N, Sabatelli P, Reggiani C, Megighian A, et al (2003) Mitochondrial dysfunction and apoptosis in myopathic mice with collagen VI deficiency. Nat Genet 35:367–371. 14625552 10.1038/ng1270

[8] Millay DP, Sargent MA, Osinska H, Baines CP, Barton ER, et al (2008) Genetic and pharmacologic inhibition of mitochondrial-dependent necrosis attenuates muscular dystrophy. Nat Med 14:442–447. 18345011 10.1038/nm1736 PMC2655270

[9] Kokoszka JE, Waymire KG, Levy SE, Sligh JE, Cai J, et al (2004) The ADP/ATP translocator is not essential for the mitochondrial permeability transition pore. Nature 427:461–465. 14749836 10.1038/nature02229 PMC3049806

[10] Krauskopf A, Eriksson O, Craigen WJ, Forte MA, Bernardi P (2006) Properties of the permeability transition in VDAC1(−/−) mitochondria. Biochim Biophys Acta 1757:590–595. 16626625 10.1016/j.bbabio.2006.02.007

[11] Baines CP, Kaiser RA, Sheiko T, Craigen WJ, Molkentin JD (2007) Voltage-dependent anion channels are dispensable for mitochondrial-dependent cell death. Nat Cell Biol 9:550–555. 17417626 10.1038/ncb1575 PMC2680246

[12] Varanyuwatana P, Halestrap AP (2012) The roles of phosphate and the phosphate carrier in the mitochondrial permeability transition pore. Mitochondrion 12:120–125. 21586347 10.1016/j.mito.2011.04.006 PMC3281194

[13] Giorgio V, von Stockum S, Antoniel M, Fabbro A, Fogolari F, et al (2013) Dimers of mitochondrial ATP synthase form the permeability transition pore. Proc Natl Acad Sci U S A 110:5887–5892. 23530243 10.1073/pnas.1217823110 PMC3625323

[14] Bonora M, Bononi A, De Marchi E, Giorgi C, Lebiedzinska M, et al (2013) Role of the c subunit of the F-O ATP synthase in mitochondrial permeability transition. Cell Cycle 12:674–683. 23343770 10.4161/cc.23599 PMC3594268

[15] Basso E, Fante L, Fowlkes J, Petronilli V, Forte MA, et al (2005) Properties of the permeability transition pore in mitochondria devoid of Cyclophilin D. J Biol Chem. 280:18558–18561. 10.1074/jbc.C500089200 15792954

[16] Ichas F, Mazat JP (1998) From calcium signaling to cell death: two conformations for the mitochondrial permeability transition pore. Switching from low- to high-conductance state. Biochim Biophys Acta 1366:33–50. 9714722 10.1016/s0005-2728(98)00119-4

[17] Brenner C, Moulin M (2012) Physiological roles of the permeability transition pore. Circ Res 111:1237–1247. 23065346 10.1161/CIRCRESAHA.112.265942

[18] Korge P, Weiss JN (1999) Thapsigargin directly induces the mitochondrial permeability transition. Eur J Biochem 265:273–280. 10491183 10.1046/j.1432-1327.1999.00724.x

[19] Blattner JR, He L, Lemasters JJ (2001) Screening assays for the mitochondrial permeability transition using a fluorescence multiwell plate reader. Anal Biochem 295:220–226. 11488625 10.1006/abio.2001.5219

[20] Picard M, Taivassalo T, Gouspillou G, Hepple RT (2011) Mitochondria: isolation, structure and function. J Physiol 589:4413–4421. 21708903 10.1113/jphysiol.2011.212712 PMC3208215

[21] Nieminen AL, Saylor AK, Tesfai SA, Herman B, Lemasters JJ (1995) Contribution of the mitochondrial permeability transition to lethal injury after exposure of hepatocytes to t-butylhydroperoxide. Biochem J 307 (Pt 1):99–106. 10.1042/bj3070099 PMC1136750 7718000

[22] Petronilli V, Miotto G, Canton M, Brini M, Colonna R, et al (1999) Transient and long-lasting openings of the mitochondrial permeability transition pore can be monitored directly in intact cells by changes in mitochondrial calcein fluorescence. Biophys J 76:725–734. 9929477 10.1016/S0006-3495(99)77239-5 PMC1300077

[23] Irwin W, Fontaine E, Agnolucci L, Penzo D, Betto R, et al (2002) Bupivacaine myotoxicity is mediated by mitochondria. J Biol Chem 277:12221–12227. 11790774 10.1074/jbc.M108938200

[24] Jones RA, Smail A, Wilson MR (2002) Detecting mitochondrial permeability transition by confocal imaging of intact cells pinocytically loaded with calcein. Eur J Biochem 269:3990–3997. 12180975 10.1046/j.1432-1033.2002.03087.x

[25] Huser J, Blatter LA (1999) Fluctuations in mitochondrial membrane potential caused by repetitive gating of the permeability transition pore. Biochem J 343:311–317. 10510294 PMC1220555

[26] Jacobson J, Duchen MR (2002) Mitochondrial oxidative stress and cell death in astrocytes–requirement for stored Ca2+ and sustained opening of the permeability transition pore. J Cell Sci 115:1175–1188. 11884517 10.1242/jcs.115.6.1175

[27] De Giorgi F, Lartigue L, Ichas F (2000) Electrical coupling and plasticity of the mitochondrial network. Cell Calcium 28:365–370. 11115375 10.1054/ceca.2000.0177

[28] Huser J, Rechenmacher CE, Blatter LA (1998) Imaging the permeability pore transition in single mitochondria. Biophys J 74:2129–2137. 9545072 10.1016/S0006-3495(98)77920-2 PMC1299554

[29] Collins CA, Zammit PS (2009) Isolation and grafting of single muscle fibres. Methods Mol Biol 482:319–330. 19089365 10.1007/978-1-59745-060-7_20

[30] Blinova K, Combs C, Kellman P, Balaban RS (2004) Fluctuation analysis of mitochondrial NADH fluorescence signals in confocal and two-photon microscopy images of living cardiac myocytes. J Microsc 213:70–75. 14678514 10.1111/j.1365-2818.2004.01278.x

[31] Manders E, Verbeek F, Aten J (1993) Measurement of colocalization of objects in dual-color confocal images. J Microsc Oxford 169:375–382. 10.1111/j.1365-2818.1993.tb03313.x 33930978

[32] Koopman WJ, Distelmaier F, Esseling JJ, Smeitink JA, Willems PH (2008) Computer-assisted live cell analysis of mitochondrial membrane potential, morphology and calcium handling. Methods 46:304–311. 18929665 10.1016/j.ymeth.2008.09.018

[33] Ward MW, Rego AC, Frenguelli BG, Nicholls DG (2000) Mitochondrial membrane potential and glutamate excitotoxicity in cultured cerebellar granule cells. J Neurosci 20:7208–7219. 11007877 10.1523/JNEUROSCI.20-19-07208.2000 PMC6772767

[34] Ricchelli F, Sileikyte J, Bernardi P (2011) Shedding light on the mitochondrial permeability transition. Biochim Biophys Acta 1807:482–490. 21377443 10.1016/j.bbabio.2011.02.012

[35] Petrat F, Pindiur S, Kirsch M, de Groot H (2003) “Mitochondrial” photochemical drugs do not release toxic amounts of 1O(2) within the mitochondrial matrix space. Arch Biochem Biophys 412:207–215. 12667484 10.1016/s0003-9861(03)00063-8

[36] Nogueira V, Devin A, Walter L, Rigoulet M, Leverve X, et al (2005) Effects of decreasing mitochondrial volume on the regulation of the permeability transition pore. J Bioenerg Biomembr 37:25–33. 15906146 10.1007/s10863-005-4120-3

[37] Alavian KN, Beutner G, Lazrove E, Sacchetti S, Park HA, et al (2014) An uncoupling channel within the c-subunit ring of the F1FO ATP synthase is the mitochondrial permeability transition pore. Proc Natl Acad Sci U S A 111:10580–10585. 24979777 10.1073/pnas.1401591111 PMC4115574

[38] Shchepina LA, Pletjushkina OY, Avetisyan AV, Bakeeva LE, Fetisova EK, et al (2002) Oligomycin, inhibitor of the F0 part of H+-ATP-synthase, suppresses the TNF-induced apoptosis. Oncogene 21:8149–8157. 12444550 10.1038/sj.onc.1206053

[39] Peachman KK, Lyles DS, Bass DA (2001) Mitochondria in eosinophils: functional role in apoptosis but not respiration. Proc Natl Acad Sci U S A 98:1717–1722. 11172017 10.1073/pnas.98.4.1717 PMC29323

